# Supplementary material for: Automated satellite remote sensing of giant kelp at the Falkland Islands (Islas Malvinas)
Source: PLoS One. 2022 Jan 6;17(1):e0257933. doi: 10.1371/journal.pone.0257933 (PMC8735600; doi:10.1371/journal.pone.0257933)
Supplement: S1 Table — Number of scenes observed during annual intervals (July-June) from the automated spectral (DTM) and citizen science (FF8) datasets. (PDF) [file pone.0257933.s001.pdf]

Annual observation days. Number of scenes observed during annual intervals (July - June) from the automated spectral (DTM) and citizen science (FF8) datasets.

Year†	DTM	FF8
1985	1	1‡
1987	5	6‡
1997	4	5
1998	6	4
1999	13	10
2000	42	12
2001	37	9
2002	49	19
2003	29	1‡
2004	47	21
2005	61	38
2006	55	22
2007	60	29
2008	33	4
2009	46	8
2010	32	0‡
2011	24	0‡
2012	33	0‡
2013	34	0‡
2014	71	46
2015	80	57
2016	70	50
2017	79	65
2018	82	26
2019	70	0‡
2020	80	0‡
2021	34	0‡

† Year corresponds to the interval spanning July of the preceding calendar year through June of the listed calendar year.

‡ FF8 years with insufficient spatial coverage of classifications were not included in the timeseries analysis.
