# Supplementary material for: Automated satellite remote sensing of giant kelp at the Falkland Islands (Islas Malvinas)
Source: PLoS One. 2022 Jan 6;17(1):e0257933. doi: 10.1371/journal.pone.0257933 (PMC8735600; doi:10.1371/journal.pone.0257933)
Supplement: S1 Fig — In situ nitrate and temperature measurements were obtained from the World Ocean Atlas within the region spanning 57.0° to 62.0°W and 50.0° to 53.0°. A synthetic nitrate model was derived from the in situ values using least-squares linear regression, and negative synthetic nitrate values were set to zero. (PDF) [file pone.0257933.s002.pdf]

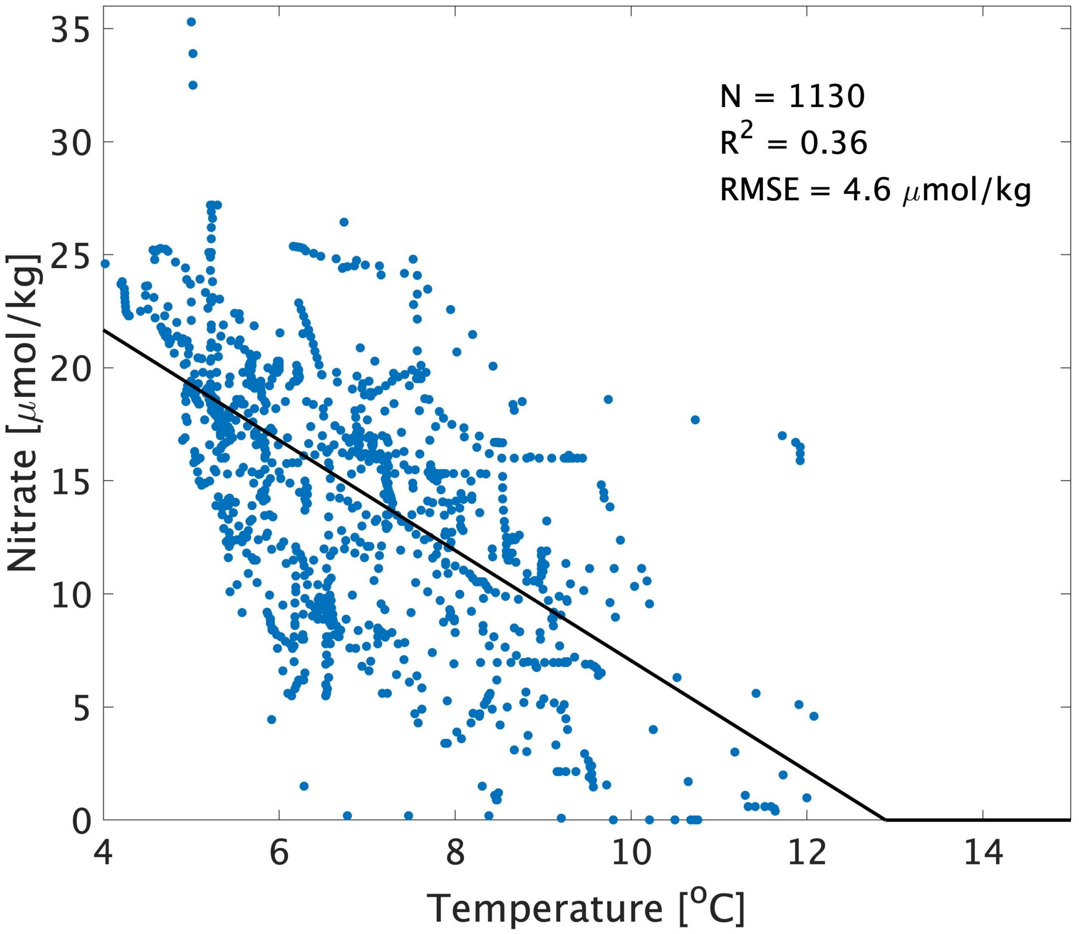

Nitrate and temperature relationship for the FLK region. Nitrate and temperature measurements obtained from the World Ocean Atlas are shown as solid blue dots, and the synthetic nitrate model is overlaid as a solid black line.
