# Supplementary material for: Automated satellite remote sensing of giant kelp at the Falkland Islands (Islas Malvinas)
Source: PLoS One. 2022 Jan 6;17(1):e0257933. doi: 10.1371/journal.pone.0257933 (PMC8735600; doi:10.1371/journal.pone.0257933)
Supplement: S2 Fig — Spatial variations in canopy trends were evaluated by aggregating annual DTM data products to their nearest 1 km coastline segments and testing for long-term trends within the data products assigned to each segment. (PDF) [file pone.0257933.s003.pdf]

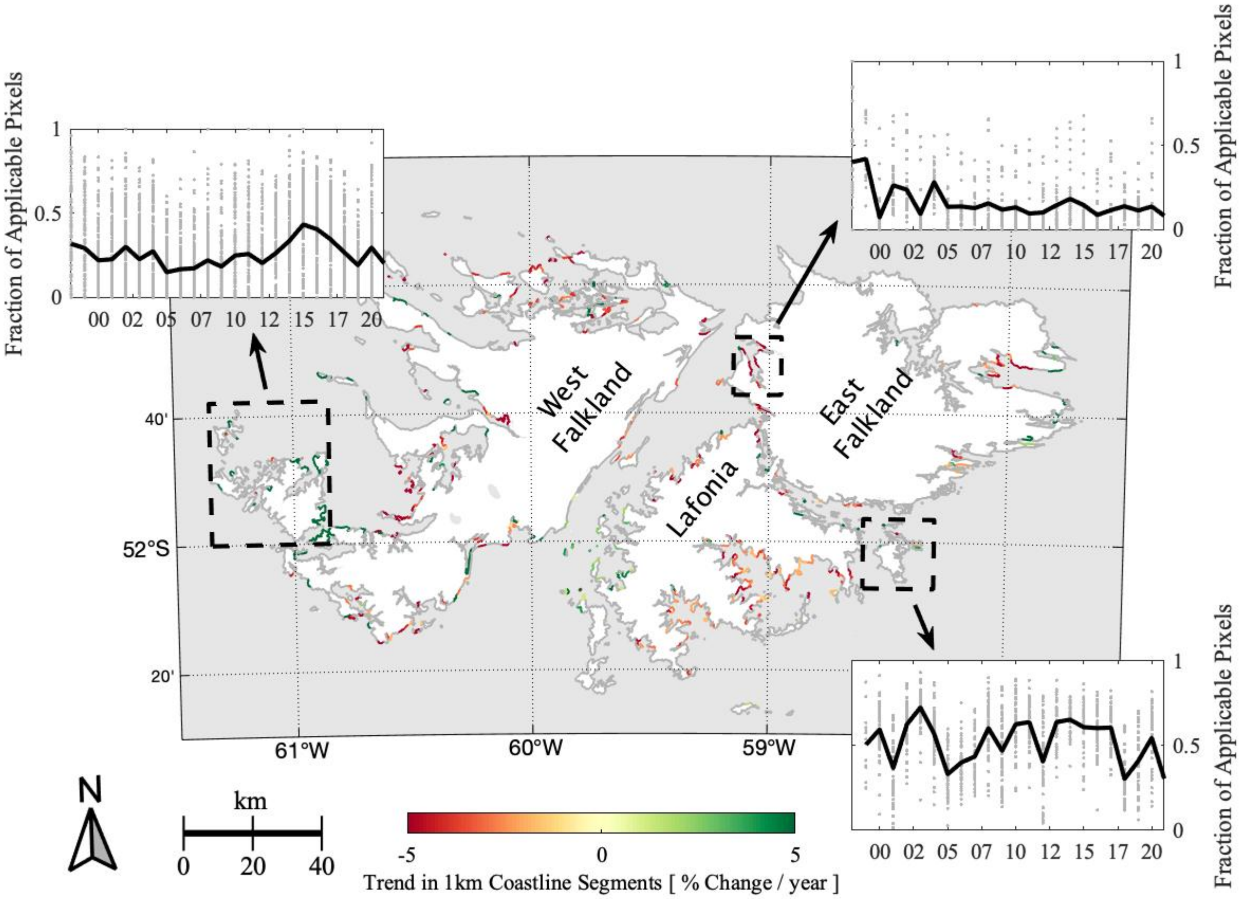

Trends in canopy extent partitioned to 1 km coastline subsets. Significant ($P < 0.01$) linear trends in annual mean canopy coverage based on DTM data products are shown in green and red. Coastline subsets wherein trends are not significant ($P \geq 0.01$) are indicated in gray. Timeseries of select coastline subsets are shown in the insets for regions indicated with a dashed black line, with year shown on the x-axis, as follows: Weddell Island and adjacent islands (upper left); San Carlos waters (upper right); and Lively Island (lower right).
